# New York Regents and the D.D.S. Degree

**Published:** 1903-01

**Authors:** 


### NEW YORK REGENTS AND THE D.D.S. DEGREE.

THE Regents of New York met at Albany, Thursday, December 4, 1902, to consider the question of transferring the M.D.S. degree to that of D.D.S. (Doctor of Dental Surgery).

The committee to whom this matter had been referred made a very able report, from which the following quotation is made: "That legislation permits the Regents to do that which the colleges either cannot or will not do, to detract the degree by the exchange proposed. For the Regents to do this would be for them to contravene their settled policy and to abuse the trust reposed in them as guardians of the degrees of the colleges over which they have supervision."

Notwithstanding this strong statement, the Regents adopted the following resolution:

> "*Resolved,* That the Regents direct the College Committee to prescribe preliminary requirements and rules for the examination of persons holding the M.D.S. degree, and that the D.D.S. degree be conferred on successful candidates who pay the prescribed fee and meet all other requirements."

While it is possible that the Regents could not give a flat refusal and keep within the law, the conclusion is one that will fail to satisfy either those in favor or in opposition, and it certainly places the Regents in a false position. If the law of the State of New York gives this body the power to confer the degree of D.D.S., then it has equally the authority to confer any degree. It seems hardly possible that such power could have been given by the Legislature with a clear comprehension of its far-reaching possibilities.

The examination required by the Regents, if made equivalent to that demanded by the dental schools of that State, will be an ordeal that few of the older men, it is surmised, will care to undergo, so that there will not be many transfers if the examination is given into the hands of those competent to make it.

To an outsider it would seem that the permission given to the Regents to confer degrees should be rescinded. It is certainly a bad law, and, while not abused by the present Board, opens a very wide door for possible future wrong-doing under a less scrupulous body.

